# Physiological ER Stress Mediates the Differentiation of Fibroblasts

**DOI:** 10.1371/journal.pone.0123578

**Published:** 2015-04-30

**Authors:** Shinsuke Matsuzaki, Toru Hiratsuka, Manabu Taniguchi, Kenta Shingaki, Tateki Kubo, Koichiro Kiya, Toshihiro Fujiwara, Shigeyuki Kanazawa, Ryutaro Kanematsu, Tameyasu Maeda, Hironori Takamura, Kohe Yamada, Ko Miyoshi, Ko Hosokawa, Masaya Tohyama, Taiichi Katayama

**Affiliations:** 1 Department of Anatomy and Neuroscience, Osaka University Graduate School of Medicine, Osaka, Japan; 2 Department of Child Development and Molecular Brain Science, United Graduate School of Child Development, Osaka University, Suita, Osaka, Japan; 3 Molecular Research Center for Children’s Mental Development, United Graduate School of Child Development, Osaka University, Suita, Osaka, Japan; 4 Department of Research & Development, Noevir Co., Ltd., Higashiomi, Shiga, Japan; 5 Department of Plastic Surgery, Osaka University Graduate School of Medicine, Osaka, Suita, Osaka, Japan; 6 Research Center for Child Mental Development, Hamamatsu University School of Medicine, Hamamatsu, Japan; 7 Division of Molecular Brain Science, Research Institute of Traditional Oriental Medicine, Kinki University, Sayama, Osaka, Japan; San Gallicano Dermatologic Institute, ITALY

## Abstract

Recently, accumulating reports have suggested the importance of endoplasmic reticulum (ER) stress signaling in the differentiation of several tissues and cells, including myoblasts and osteoblasts. Secretory cells are easily subjected to ER stress during maturation of their secreted proteins. Skin fibroblasts produce and release several proteins, such as collagens, matrix metalloproteinases (MMPs), the tissue inhibitors of metalloproteinases (TIMPs) and glycosaminoglycans (GAGs), and the production of these proteins is increased at wound sites. Differentiation of fibroblasts into myofibroblasts is one of the key factors for wound healing and that TGF-β can induce fibroblast differentiation into myofibroblasts, which express α-smooth muscle actin. Well-differentiated myofibroblasts show increased production of collagen and TGF-β, and bring about wound healing. In this study, we examined the effects of ER stress signaling on the differentiation of fibroblasts, which is required for wound healing, using constitutively ER stress-activated primary cultured fibroblasts. The cells expressed positive α-smooth muscle actin signals without TGF-β stimulation compared with control fibroblasts. Gel-contraction assays suggested that ER stress-treated primary fibroblasts caused stronger shrinkage of collagen gels than control cells. These results suggest that ER stress signaling could accelerate the differentiation of fibroblasts to myofibroblasts at injured sites. The present findings may provide important insights for developing therapies to improve wound healing.

## Introduction

The endoplasmic reticulum (ER) is an organelle observed in eukaryotic cells. Protein folding occurs in the ER before the proteins are transported to the extracellular surface or intracellular organelles. Several cellular stress conditions that can lead to accumulation of unfolded or misfolded proteins in the ER lumen are collectively called ER stress [1, 2]. Eukaryotic cells have a system to overcome cellular damage induced by ER stress, which is termed the unfolded protein response (UPR). In mammals, three ER stress transducers, namely PKR-like endoplasmic reticulum kinase, inositol-requiring 1, and activating transcription factor 6, play important roles in UPR signal transduction. However, once ER stress exceeds the UPR system, apoptotic signals appear in cells under ER stress.

Apoptosis is an active process of cell death that is essential for successful organogenesis during development and normal physiological homeostasis throughout adulthood, and is often associated with cell differentiation [3–6]. ER stress can activate rodent caspase-12 and human caspase-4, which are members of a family of cysteine proteases, resulting in the induction of apoptosis [7, 8]. Such apoptosis related to ER stress is clearly observed in the pathology of misfolded protein-related diseases, such as Alzheimer’s disease, Parkinson’s disease, and prion disease [9–11].

However, previous reports have suggested that ER stress is also present under physiological conditions, such as the early stages of myoblast differentiation [6]. Not only apoptotic cells, but also differentiating myoblasts showed induction of two ER stress-specific proteins: CHOP, a transcription factor related to ER stress-induced apoptosis, and BiP/GRP78, an ER-specific molecular chaperone. Inhibition of ATF6 activation by ER stress was found to block apoptosis and myotube formation in culture models of myogenesis [6]. These studies raise the possibility that ER stress or ER stress signaling is required under physiological conditions to induce differentiation of cells.

In the wound healing process, fibroblasts infiltrate into the damaged area where they proliferate and differentiate into myofibroblasts. In normal wound healing in humans, myofibroblasts disappear from the scar once the tissue integrity has been restored [12–14]. However, in abnormal healing and fibrocontractile diseases, myofibroblasts persist in the tissue. The presence of these cells has been noted in actively contracting granulation tissue and hypertrophic scars [15, 16], as well as in contractile tissues such as the palmar fascia in Dupuytren’s disease [17, 18]. These reports suggest that myofibroblasts are one of the key factors for tissue contraction and fibrosis. Furthermore, a recent study showed that ER stress signaling is involved in keloid scar formation [19], in which myofibroblasts should work actively, suggesting that ER stress affects actively contracting granulation tissue and hypertrophic scars via myofibroblasts.

Myofibroblasts show characteristics of both fibroblasts, including extensive rough ER and Golgi apparatus, and smooth muscle cells, including an extensive cytoplasmic microfilamentous apparatus of α-smooth muscle actin (α-SMA), the actin isoform typical of vascular smooth muscle cells. Previous studies have established that normal dermal fibroblasts respond to TGF-β1 treatment by increasing their α-SMA content [20] and contractile capacity [21] and that IFN-γ treatment causes both parameters to decrease. [22, 23]

During wound healing, fibroblasts are required to synthesize and secrete abundant proteins. It is well-known that ER stress signals are detected in secretory cells such as chondrocytes [24] and pancreatic β-cells [25, 26] and that ER stress plays an important role in the differentiation of chondrocytes and myoblasts. [27] Thus, we hypothesized that fibroblasts in the wound healing process are exposed to ER stress, resulting in the acceleration of fibroblast differentiation into myofibroblasts. In this study, we examined the effects of repeated ER stress exposure on the differentiation of fibroblasts and evaluated whether physiological ER stress is involved in the wound healing system.

## Results

**Fig 1:**
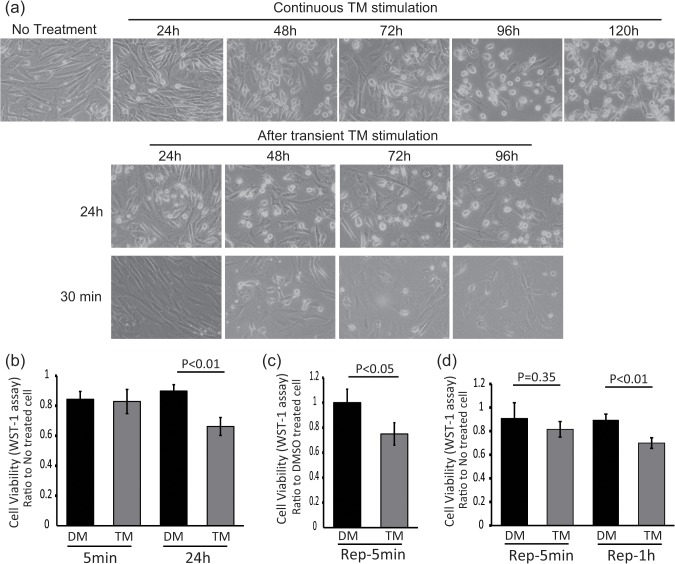
Condition setteing for physiological ER stressed fibroblast. (a) (upper panels) Primary cultured fibroblasts were treated with 1μg/ml TM. The cells were observed each time points. (middle and bottom panels) Primary cultured fibroblasts were transiently treated with 1μg/ml TM for 24 h or 30 min and the medium were changed to the culture medium. The cells were observed each time points after the medium change. (b-d) The effect of each ER stress methods on cell viability was measured by WST-1 assay. (b) 24h or 5 min of transient 2μg/ml TM stimulation, (c) 5 min of repeated 2μg/ml TM stimulation and (d) 1h or 5 min of repeated 1μg/ml TM stimulation were adopted for these assays. Same amount of DMSO were used as controls. The P value was compared with the control and calculated by Student's T test.

Recent studies have suggested the importance of physiological roles of ER stress in the differentiation of myoblasts and osteoblasts. Thus, we hypothesized that physiological ER stress could mediate the differentiation of fibroblasts. To determine the conditions for collecting primary fibroblasts under physiological ER stress, we examined several culture methods (Fig 1). Continuous 1μg/ml tunicamycin (TM) stimulation induced higher mortality levels for primary fibroblasts at 24, 48, 72, and 96 h than 24h or 30 min of transient 1μg/ml TM stimulation (Fig 1A). However, 24 h of transient 1μg/ml TM stimulation caused mild mortality after 24h, and 30 min of 1μg/ml transient TM stimulation induced mild mortality after 48 h (Fig 1A, middle and bottom). Then, to examine the effect of TM on cell death precisely, we measured cell viability under several TM stimulation by WST-1 assay (Fig 1B, 1C and 1D). 24h of transient 2μg/ml TM stimulation caused significant reduction of cell viability, but 5 min of transient 2μg/ml TM stimulation did not (Fig 1B). However, the cells treated with 5 min of 2μg/ml TM stimulation repeated three times every 24h reduced cell viability significantly (Fig 1C). Finally, 5 min or 1h of 1μg/ml TM stimulation repeated three times every 24h were attempted. 1h of repeated 1μg/ml TM stimulation caused significant reduction of cell viability, but 5 min of transient 1μg/ml TM stimulation did not (Fig 1D). Thus, we decided that our conditions for obtaining ER-stressed primary fibroblasts were 5 min of TM stimulation repeated three times every 24h.

**Fig 2:**
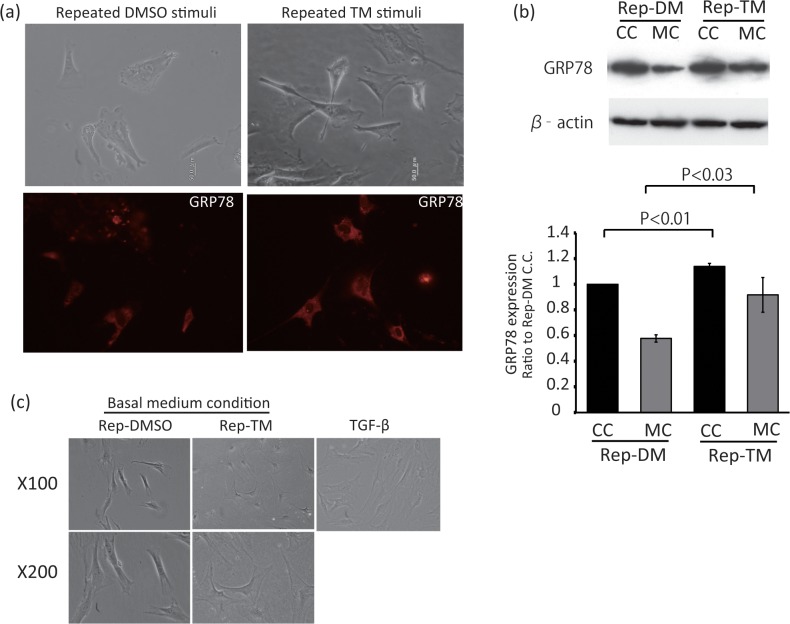
Effects of repeated TM stimulation on fibroblasts’ morphology and GRP78/BiP expression. Primary cultured fibroblasts were treated with 1μg/ml TM or DMSO for 5 minutes per day 3 days in series. After this repeated TM or DMSO stimulation, medium was changed to DMEM with 2% horse serum (Basal medium condition) and incubated for 12h to induce differentiation. Primary cultured fibroblasts were treated with 1μg/ml TM or DMSO for 5 minutes per day 3 days in series. (a) Just after this repeated TM or DMSO stimulation, the cells were observed (upper panels) and stained by anti-Bip antibody (bottom panels). (b) The cells treated with TM (Rep-TM) or DMSO (Rep-DM/Rep-DMSO) cultured in the culture condition medium (C.C.) or in the Basal medium condition for differentiation (M.C.) were collected and lysed. Western blot analysis was performed using an anti-Bip or anti-β-actin primary antibody (upper panels). Quantitative data were obtained by densitometry of the bands. Data are expressed as the mean ± SEM for at least three independent experiments (shown as a ratio of the Rep-DM C.C.). The P value was compared with the control and calculated by Student's T test. (c) Left and middle panels show the cells treated with TM (Rep-TM) or DMSO (Rep-DMSO) cultured at Basal medium condition. Right panel shows the cells treated with TGF-β1 after the incubation at the basal medium condition.

To examine the effect of repeated 5 min TM stimulation of ER stress pathway, BiP/GRP78 levels were detected by immunocytochemistry and western blot analysis. 24h after the final stimulation, ER-stressed primary fibroblasts showed higher BiP/GRP78 levels than control cells under the culture conditions, but did not show any morphological differences (Fig 2A and 2B). Even under the basal medium condition, which means more than 36h after the final stimulation, ER-stressed primary fibroblasts showed higher BiP/GRP78 levels than control cells under the culture conditions. These data suggest that ER-stressed primary fibroblasts have resistance to ER stressors and an activated ER stress response pathway.

**Fig 3:**
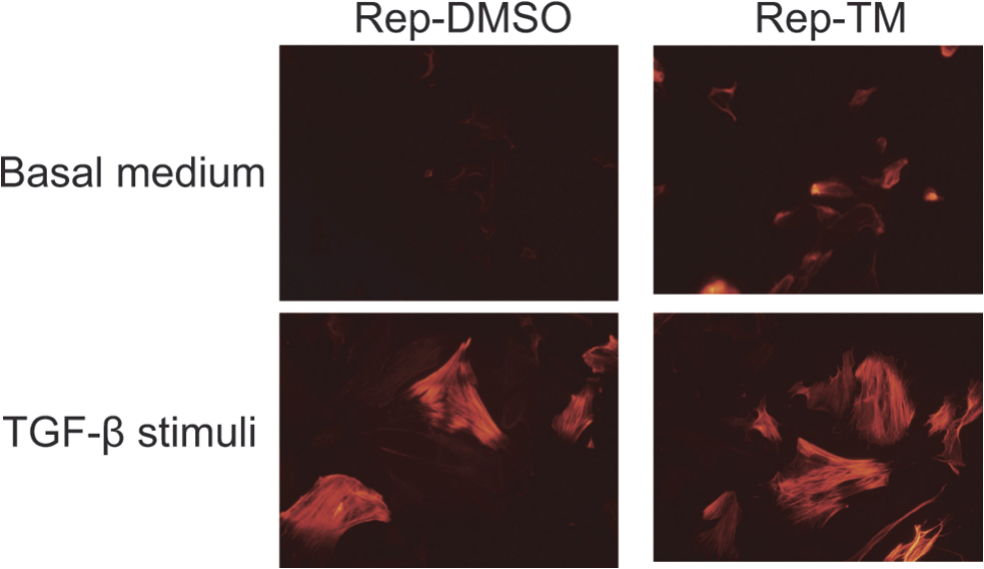
The repeated TM treated fibroblasts showed α-SMA induction. Rep-TM and Rep-DMSO cells were cultured at basal medium for 12 h (upper panels) and each cells were treated with TGF-β1 after basal medium condition for 48 h. The cells were fixed and immunocytochemistry was performed with anti- α-SMA antibody.

It is well-known that fibroblasts differentiate into myofibroblasts and release several kinds of cytokines, and that this differentiation is very important for wound healing. [28] Therefore, as a next step, we examined the effects of ER stress signals on the differentiation of fibroblasts. As mentioned before, ER-stressed primary fibroblasts did not show any marked differences compared with control fibroblasts (Fig 2A). TGF-β stimulation caused differentiation of fibroblasts into myofibroblasts, which showed wide spreading of the cytosol and high levels of α-SMA expression (Fig 2C, right panel; Fig 3, bottom panels). However, after 3 days of culture in basal medium, ER-stressed fibroblasts showed myofibroblast-like changes, including wide spreading of the cytosol, while control fibroblasts did not (Fig 2, middle panel). To confirm the differentiation to myofibroblasts, the expression levels of α-SMA were examined by ICH. ER-stressed fibroblasts showed higher α-SMA levels than control cells, but lower levels than TGF-β-treated fibroblasts (Fig 3).

**Fig 4:**
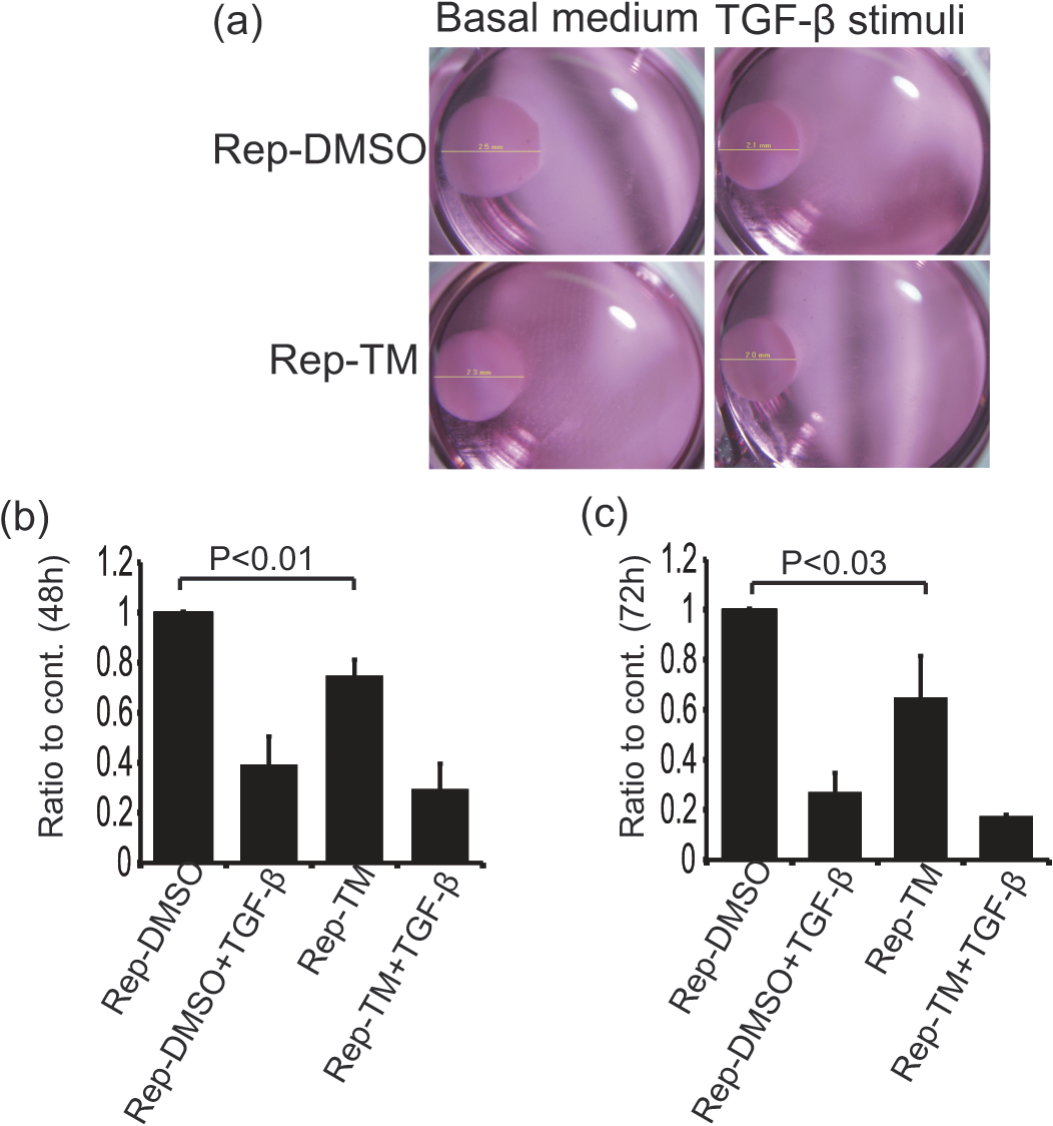
The repeated TM treated fibroblasts showed accelerated collagen gel contraction. Rep-TM and Rep-DMSO cells were embedded in collagen gels. After gel formation, the rims of the gels were detached and gels were overlaid with basal medium or TGF-β1 containing medium. (a) these pictures show one of the experimental results of gel contraction. (b and c) Gel size of each conditions were measured at 48 and 72 h. Gel size containing Rep-DMSO cells treated with basal medium were used as control. Each bars show the ratio to control at 48 h (b) and 72 h (c). The P value was compared with the control and calculated by Student's T test.

Myofibroblasts, which are well-differentiated fibroblasts, are important for the formation of elastic areas of skin, and normally show a stronger shrinkage potential that is effective for wound healing. Therefore, we performed collagen gel assays using ER-stressed primary fibroblasts. As control cells, primary fibroblasts were cultured in collagen gels, and the size changes of the collagen gels in basal medium were examined to show the shrinkage power of these cells (Fig 4A, upper left panel). The control cells showed reasonably smaller collagen gel sizes after treatment with TGF-β, reflecting the effects of myofibroblasts (Fig 4A, upper right panel; Fig 4B and 4C). According to the level of α-SMA expression, ER-stressed primary fibroblasts showed significantly smaller collagen gel sizes compared with control primary fibroblasts without TGF-β, as the basal condition (Fig 4). The TM-treated cells tended to show stronger effects than the control fibroblasts under TGF-β treatment, but not significantly (Fig 4). These data suggest that physiological level of mild ER stress-induced differentiation of fibroblasts and contribute to wound healing processes.

## Discussion

In this study, to produce fibroblasts that can mimic the cells under the physiological ER stress observed at wound healing sites, we used repeated TM stimulation. The TM-treated fibroblasts showed a different morphology, with wide spreading of the cytoplasm, and increased α-SMA expression in the basal medium. In addition, the cells treated with TM showed stronger shrinkage of collagen gels. These findings suggest that fibroblasts undergo facilitated differentiation into myofibroblasts under physiological ER stress. This indicates that fibroblasts under physiological ER stress at wound healing sites could easily differentiate into myoblasts to recover the wound area because of protein synthesis and secretion.

### Physiological ER stress at wounded areas and differentiation

Fibroblasts are connective tissue cells that secrete an extracellular matrix rich in collagens and other macromolecules. During wound healing, fibroblasts are required to synthesize and secrete abundant proteins including collagens, elastin, matrix metalloproteinases (MMPs), the tissue inhibitors of metalloproteinases (TIMPs) and glycosaminoglycans (GAGs) [29]. High demand of protein synthesis can cause ER stress especially in secretory cells such as chondrocytes [24] and pancreatic β-cells [25, 26] and that ER stress plays an important role in the differentiation of chondrocytes [24] and myoblasts [6]. Thus, the wound healing process could be a source of ER stress and a trigger for the differentiation of fibroblasts to myofibroblasts.

In this study, we treated skin fibroblasts with TM repetitively to produce cells mimicking fibroblasts under mild ER stress at wound healing areas. As shown in Fig 1, the cells showed high viability and increased GRP78 expression, even when the medium changed to the culture medium. Thus, we considered that the repetitively-treated fibroblasts could be used as cells mimicking fibroblasts at wound areas.

Previous studies have shown the importance of ER stress for the differentiation of chondrocytes [24], plasma cells [30, 31], myoblasts [6], and other cell types [27, 32–35]. The cells expressed ER stress markers during their differentiation, and knockout of certain ER stress pathway signaling components could inhibit the differentiation. For example, Xbp1 gene deletion caused abnormal differentiation of plasma cells [30, 31] and Bbf2h7 knockout disturbed chondrocyte differentiation [24]. In the present study, TM-treated fibroblasts were compared with DMSO-treated fibroblasts to examine the effects of ER stress on the differentiation of fibroblasts. Normally, TGF-β is used to induce myofibroblasts and we needed to change the medium to a basal medium containing a low level of serum as preparation before TGF-β stimulation. As shown in Fig 2, after culture in the basal medium, the TM-treated fibroblasts showed wide spreading of the cytoplasm compared with DMSO-treated fibroblasts, even though DMSO-treated fibroblasts showed the same morphology to non-treated fibroblasts under these culture conditions. In addition, the TM-treated cells expressed higher levels of α-SMA and caused stronger gel contraction capacity than the DMSO-treated cells under the basal medium conditions. These data suggest that fibroblasts with activated ER stress pathways are more sensitive to the differentiation switch, similar to the case for chondrocytes and plasma cells [24, 30, 31]. However, it is also reported that while ER stress inducers promoted apoptosis during myoblast fusion, the survived cells exhibited high resistance against the apoptotic stimuli [6], resulting inefficient formation of contracting myofibers. In other words, the cell death induced by ER stress may serve to selectively produce fibroblasts sensitive to the differentiation signals, although we did not detect any remarkable cell death after application of ER stress stimuli. Nevertheless, we obtained fibroblasts that showed a tendency to differentiate into myofibroblasts, and thereafter examined the effects of TGF-β on TM- or DMSO-treated fibroblasts. According to the results observed in the basal medium, the TM-treated cells showed a tendency for stronger gel contraction than the DMSO-treated cells after TGF-β stimulation. The effects of TGF-β on the differentiation of fibroblasts should be sufficiently strong, meaning that TGF-β stimulation should cover the acceleration in fibroblast differentiation caused by ER stress. Thus, mild ER stress should be involved in the differentiation of fibroblasts, and UPR signaling may be essential for their differentiation.

### Possible involvement of ER stress in dermal diseases

Previous studies have indicated the involvement of ER stress in dermal disorders such as keloid scar formation [19] and recessive cutis laxa [36]. Previous reports have also shown that hypoxic conditions can impair fibroblast differentiation into myofibroblasts [37]. In recessive cutis laxa, reduced extracellular matrix formation was observed under ER stress induced by mutation of fibulin-5 [36]. However, enhanced extracellular matrix formation resulted in keloid scar formation. These reports indicated that exquisite control of the degree of ER stress should be mandatory for the maintenance and recovery of skin tissue. For clear understanding of the bimodal function of ER stress, it will be helpful to consider the selection of surviving cells in muscles and apoptotic cells under ER stress. Once myoblasts survive ER stress stimuli, they acquire resistance to the apoptosis caused by ER stress and show efficient differentiation into contracting myofibers [6]. Conversely, excessive ER stress can cause apoptosis and loss of many cells in tissues, thereby leading to a shortage of cells to maintain the tissue. In our case, mild ER stress should select or change the characteristics of fibroblasts and may accelerate their differentiation into myoblasts.

Our findings suggest one of the mechanisms for wound healing from the viewpoint of ER stress and partially explains the pathology for keloid scar formation. These observations may provide important insights for the development of therapies to promote wound healing.

## Materials and Methods

### Primary Cell Culture

Skins were dissected from the back of 2-day-old C57B6 mice deeply anesthetized with sodium pentobarbital and explanted on plastic dishes. Skin fibroblasts were cultured in Dulbecco's modified Eagle's medium (DMEM) containing 10% fetal bovine serum. Cultures were incubated at 37°C in a humidified atmosphere of 5% CO2/95% air. To investigate the effect of ER stress on fibroblasts, cells at passage 2 were treated with 1μg/ml tunicamycin, an inhibitor of N-glycosylation in the ER, or dimethyl sulfoxide (DMSO) for 5 minutes per day 3 days in series. Then at passage 3, medium was changed to DMEM with 2% horse serum and incubated for 12h to induce differentiation. After this treatment, medium was returned to DMEM with 2% horse serum with or without 10ng/ml TGF-β1 (R&D Systems Inc.). All experiments were carried out in accordance with a protocol approved by the Institutional Animal Care and Use Committee of Osaka University.

### Cell viability assay by WST-1 activity

Primary fibroblasts (1×10^4^) were plated onto 96-well plates 36 h before DMSO or TM treatment. The time points to measure the cell viability were shown in supplemental data. Following insult exposure, cells were washed twice with phosphate buffered saline (PBS) and cultured with DMEM (D1145, SIGMA) and WST-1 mixed medium for 3 hours. WST-1 was measured at an absorption of λ450 nm—λ650 nm. Data are expressed as the mean ± SEM for at least three independent experiments.

### Western blot analysis

Treated cells were washed twice with PBS, harvested and lysed in RIPA buffer and protease inhibitor cocktail (Roche, Sydney, Australia). Equal amounts of protein were subjected to 5–20% gradient SDS-PAGE, e-PAGEL (ATTO CO., Tokyo, Japan) for GRP78/Bip or β-actin and transferred to PVDF membrane (Millipore, Bedford, MA). The membrane was blocked with 5% (w/v) skim milk. The membranes were incubated with a mouse anti-BiP/GRP78 antibody (BD Transduction Laboratories) followed by incubation with an HRP-conjugated anti–mouse IgG antibody (Cell Signaling Technology) to detect BiP/GRP, or they were incubated with anti-β-Actin HRP-DirecT (MBL Co., Nagoya, Japan) for β-actin. Proteins were visualized with a Luminata Classico Western HRP substrate (MERCK MILLIPORE). Quantitative data were obtained by densitometry by using ImageJ. Data are expressed as the mean ± SEM for at least three independent experiments.

### Collagen Gel Contraction Assay

To correlate tunicamycin-treatment of fibroblast cells and the contractile potential for collagen substrate, approximately 2×10^5^ cells were embedded in collagen matrix using Collagen Gel Culturing Kit (Nitta Gelatin, Inc.) as follows. Cells were mixed with reconstituted collagen solution, which consists of eight volumes of type I collagen solution and one volume of reconstituted buffer (50mM NaOH 260mM NaHCO_3_ 200mM HEPES) on ice. Then 0.5 ml aliquots of this reconstituted collagen solution were placed on the bottom of 4-well culture plates and immediately warmed at 37°C to allow gel formation. After gel formation, the rims of the gels were detached and gels were overlaid with DMEM supplemented with 2% horse serum. The cell size were measured by Image J. Data are expressed as the mean ± SEM for at least three independent experiments.

### Immunocytochemistry

Fibroblast cells were washed twice by PBS and fixed with 4% paraformaldehyde. Cells were blocked with 5% BSA and 0.1% triton-X, and incubated with the rabbit monoclonal antibody for α-SMA (Epitomics, Inc., CA, USA) or rabbit polyclonal antibody for Bip (Cell Signaling Technology, Beverly, MA), followed by incubation with secondary fluorescein isothiocyanate (FITC)-conjugated anti-rabbit IgG antibody. Confocal microscopy was performed using an LSM-510 laser scanning microscope (Carl Zeiss, Oberkochen, Germany)

## Supporting Information

S1 Fig Schema of our experimental time course. (upper panels) The methods for cell viability assay. (bottom panels) the methods for the other experiments.
(EPS)
